# Durability Variation Among Medical Gloves Made from Existing and New Elastomers Poses a Risk to Public Health

**DOI:** 10.1002/gch2.202300100

**Published:** 2023-08-15

**Authors:** Ashley Herkins, Katrina Cornish

**Affiliations:** ^1^ Department of Food, Agricultural and Biological Engineering The Ohio State University 590 Woody Hayes Drive Columbus OH 43210 USA; ^2^ Department of Horticulture and Crop Science The Ohio State University 1680 Madison Avenue Wooster OH 44691 USA

**Keywords:** elastomers, glove durability, guayule latex, medical gloves

## Abstract

Despite being an essential line of defense in preventing the spread of diseases, medical glove durability is neither measured routinely nor has standard specifications. In this study, a new glove durability assessment device is used to objectively compare the durability of gloves made of a variety of elastomers from different manufacturers. Results are related to several mechanical tests, including stress relaxation, tensile and tear tests. Overall, natural latex gloves far outperformed those made of synthetic elastomers, and there is great disparity among the different brands of nitrile gloves, some of which do not meet nitrile glove performance requirements. The study includes prototype gloves made from guayule latex, a domestic source of alternative natural rubber latex, currently under commercial development. The guayule gloves outperformed all other gloves tested, including those made from Hevea latex, without posing allergy risks. Mechanical analysis demonstrated that the guayule gloves are as strong as the best alternatives, are softer and more elastic, have better tear strength, and have such low stress relaxation that they cause very little hand fatigue during use. Guayule latex can address the need for domestic production of gloves to resolve supply chain and quality issues and encourage a shift back to natural latex gloves, which will significantly diversify the natural rubber supply.

## Graphical abstract

Medical glove durability and mechanical performance varies greatly depending on material, manufacturer, thickness, and function (examination or surgical). This study is intended to alert healthcare workers about the true quality of both existing and new glove varieties so that they can avoid flimsy gloves that easily puncture and expose them to harmful pathogens.

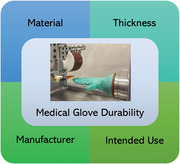

## 1 Introduction

Healthcare workers rely on medical gloves to protect them and their patients against illness‐inducing pathogens such as bacteria and viruses as well as patient contamination with dangerous drugs like fentanyl.^[^ ^1^ ^]^ Medical gloves provide a physical barrier against blood and other bodily fluids, which may contain harmful pathogens. Unfortunately, many of these gloves may be providing a false sense of security due to lax manufacturing standards and inadequate inspection rates. One study found a 33% leak rate (n = 679) among post‐use latex surgical gloves and a 5.5% defect rate among unused latex surgical gloves, which exceeds the allowable U.S. Food and Drug Administration (FDA) defect rate of 1.5%.^[^ ^2^ ^]^

While the American Society for Testing and Materials (ASTM) does provide general standards for surgical and examination gloves, it does not provide a standard for glove durability once the gloves are removed from their packaging and donned. ASTM's water inflation test for the detection of holes in medical gloves, usually used to test samples of gloves before they are packaged, can be used to check gloves after they have been worn, but this is not useful as a durability measure without a large amount of use data.^[^ ^3^, ^4^, ^5^ ^]^ Additionally, ASTM's minimum physical requirements for synthetic gloves such as nitrile, poly(vinyl chloride) (PVC), and polychloroprene were lowered from the general standards for examination gloves due to the underperformance of these materials. Without a durability standard, some glove manufacturers may produce low quality, easily damaged gloves to maximize profits.

Previous studies have demonstrated that glove durability also depends heavily on the material and composition (such as filler loading) of the glove, with natural latex outperforming synthetic materials.^[^ ^6^, ^7^, ^8^, ^9^ ^]^ Natural latex gloves are also more elastic, softer, equally as strong as premium synthetics, and have lower stress relaxation, allowing extended use without significant hand fatigue. However, the widespread occurrence in the 1990's of life‐threatening Type I latex allergies, caused by high levels of soluble proteins in gloves not properly washed during manufacture, led to a shift away from natural latex gloves to petroleum‐derived synthetic gloves. Today, ≈62.5% of the global medical glove market is synthetic.^[^ ^10^ ^]^ The poorer performance of synthetic gloves forced the FDA to approve such gloves for sale under the premise that any glove is better than no glove to control HIV/AIDS transmission. These gloves could not meet the ASTM standards for natural latex gloves, and ASTM established lower standards for the synthetics to accommodate the FDA.^[^ ^3^, ^4^ ^]^

Glove durability also reflects the ability of the specific manufacturer, meaning that two gloves of the same material and specification can have vastly different mechanical and in‐use performance.^[^ ^7^, ^8^ ^]^ This large variation in glove quality combined with the lack of an ASTM durability standard led to the invention of a glove durability assessment device.^[^ ^11^ ^]^ The device works by creating a vacuum within the base of a prosthetic hand, upon that a glove is donned, and repeatedly moving a sandpaper‐covered roller into contact with the fingertips of the hand, which are made of porous mufflers.^[^ ^11^ ^]^ The durability of the glove is quantified based on the number of sandpaper touches the glove withstands before perforating.

This study compares the relative durability of dry examination and surgeon's gloves made from a variety of elastomeric materials, both natural and synthetic, and by different manufacturers. Prototype surgical gloves made from guayule latex were also included. This new material is under commercial development and so was assessed in parallel with the existing glove materials.

## 2 Durability Testing Devices

### 2.1 Capstone Glove Assessment Device (C‐GAD)

The original iteration of the glove durability assessment device (C‐GAD) was created by senior engineering students at the Ohio State University as part of a capstone design project.^[^ ^4^ ^]^ The main drawback of the C‐GAD was that the gloves needed to be visually inspected for perforations while the testing device was stopped, which reduced the precision of results to a minimum inspection interval of 5 s.^[^ ^11^ ^]^

### 2.2 New Glove Assessment Device (N‐GAD)

The N‐GAD was built to improve upon the C‐GAD prototype. It is fully mechanized and automatically detects glove breakage immediately, eliminating the need for visual glove inspection. It is also more controllable, with adjustable settings for roller force and motor speed (touch interval), and has a liquid spray system that allows the user to simulate glove use in wet environments, although this function was not used in the current study.^[^ ^11^ ^]^

## 3 Results

### 3.1 N‐GAD and C‐GAD Comparison

Although the collected data included both time to failure and number of sandpaper touches to failure, the statistical analyses focused only on the number of touches because the time interval between touches occasionally varied. The N‐GAD was programmed to maintain a pressure differential by repeatedly de‐pressurizing throughout the test while the timer remained on, and so the time and number of touches did not directly correspond with one another.

**Figure 1:**
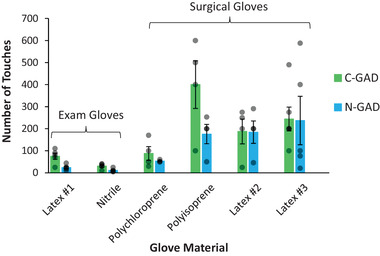
Comparison of glove durability assessed by two durability testing devices. Each value is the mean of 3 to 5 samples ± standard error. The individual points are also plotted (gray circles).

The number of touches to failure was similar with both devices except for the polyisoprene surgical glove (Sensicare Micro) (**Figure** **1**). The higher C‐GAD mean is likely due to the lower accuracy of manual glove inspection because very small perforations are often difficult to see and may be missed, artificially increasing the overall average number of touches to obvious failure. The N‐GAD eliminates human error from the glove durability testing process.

No significant difference was observed between the results from the two devices (**Table** **1**) and there was no interaction between glove type and device. However, the different gloves did have significantly different durability (*p* < 0.0001). The variability within the replicates was very high. Surgeon's gloves “latex #3” and the polyisoprene version, had gloves which were among the most and least durable of those tested.

**Table 1:** Two‐way analysis of variance comparing glove failure rates on the C‐GAD and N‐GAD. Note that α = 0.05

Source of Variation	Degrees of Freedom	Sum of Squares	F Ratio	Probability
Glove Brand	5	558 806	8.115	<0.0001
Device	1	41 798	3.035	0.089
Glove Brand x Device	5	72 774	1.057	0.398

### 3.2 Durability of a Broad Range of Gloves

**Figure 2:**
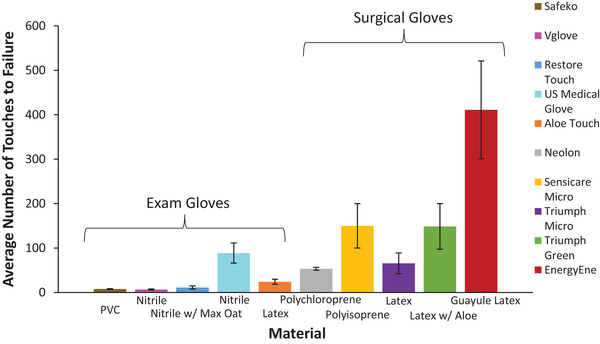
Average number of sandpaper touches to glove failure ± standard error. Examination gloves are the mean of 5 samples, and surgical gloves are the mean of 3 samples.

When the N‐GAD was used to quantify the durability of a wide variety of glove materials and brands (**Figure** **2**), a one‐way analysis of variance (α = 0.05) indicated that their durability significantly differed (*p* < 0.0001) (**Table** **2**). Student's *t*‐tests determined statistically significant glove‐to‐glove differences and indicated that the EnergyEne brand prototype guayule latex gloves were significantly more durable than all other gloves tested (**Table** **3**, Figure 2). The two glove brands with the lowest averages, Vglove and Safeko, were statistically different from the four glove brands with the highest averages, EnergyEne, Sensicare Micro, Triumph Green, and U.S. Medical Glove (Table 3).

**Table 2:** One‐way analysis of variance comparing glove failure rates for various glove types using the N‐GAD. Note that α = 0.05

Source of Variation	Degrees of Freedom	Sum of Squares	Mean Square	F Ratio	Probability
Glove Brand	9	452746	50305	12.8	<0.0001
Error	30	117934	3931	–	–
Total	39	570680	–	–	–

**Table 3:** Student's *t*‐test comparing glove failure rates for various glove types using the N‐GAD. Different letters indicate significantly different durability. Examination gloves are the mean of 5 samples, and surgical gloves are the mean of 3 samples

Connecting Letters Report					
Brand					Mean
EnergyEne	A				411.00
Sensicare Micro		B			150.00
Triumph Green		B			148.67
U.S. Medical Glove		B	C		88.80
Triumph Micro		B	C	D	65.67
Neolon		B	C	D	53.33
Aloe Touch			C	D	24.20
Restore Touch			C	D	11.20
Safeko				D	7.80
Vglove				D	6.80

The durability variability within individual glove types is also apparent between gloves manufactured by different companies from the same material (Figure 2). For example, among the three nitrile examination glove brands tested only the US Medical Glove product was reasonably durable and far outlasted the other nitrile gloves as well as the thin Aloe Touch natural latex glove. The Vglove was the worst glove tested, and user experience (Cornish laboratory) of how quickly this glove fails when donned (within a few seconds) matches the test data.

### 3.3 Glove Thickness and Durability

**Figure 3:**
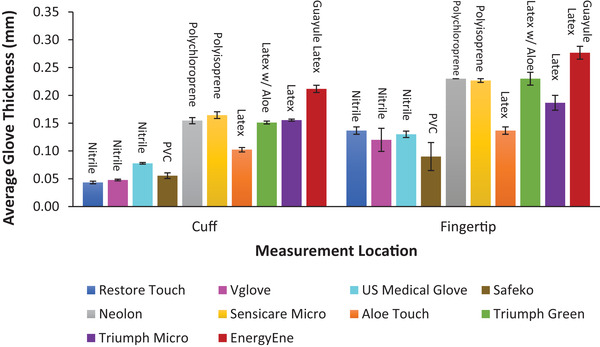
Average thickness of various glove types at the cuff and fingertip ± standard error. Four thickness measurements were collected from three replicate gloves.

**Figure 4:**
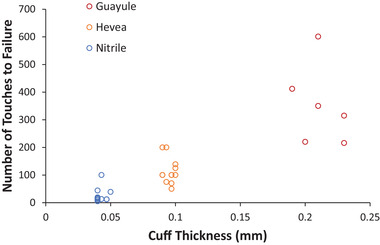
Number of touches to failure versus glove cuff thickness for Hevea latex, synthetic nitrile, and guayule latex gloves.

Gloves tend to be thicker at the fingertips than at the cuff, and thickness varies depending on material and manufacturer (**Figure** **3**). The same data are represented in the form of the average ratio of cuff thickness to fingertip thickness (**Table** **4**), and the data are also ranked in numerical order. In general, thicker gloves are more durable, regardless of the material. Thicker gloves also need more abrasive contact before they puncture (**Figure** **4**), meaning that they are a more effective barrier against harmful pathogens.

**Table 4:** Average ratio of cuff thickness to fingertip thickness based on three samples of each glove variety + standard error. Data are ranked from lowest ratio to highest ratio. Neolon brand does not have a standard error ratio because the standard error for the fingertip measurements was 0

Brand	Average Ratio of Cuff Thickness to Fingertip Thickness
Vglove	0.42 + 0.07
Restore Touch	0.51 + 0.35
US Medical Glove	0.60 + 0.25
EnergyEne	0.65 + 0.57
Triumph Green	0.66 + 0.23
Neolon	0.67 + 0.00
Safeko	0.70 + 0.20
Sensicare Micro	0.72 + 1.81
Aloe Touch	0.75 + 0.60
Triumph Micro	0.84 + 0.13

The large variation in the number of touches to failure for guayule latex gloves is due to each guayule glove being individually dipped. Also, different dwell times were used to change thickness. These result in less‐consistent durability between these prototype gloves than mass‐manufactured gloves.

### 3.4 Force Testing

**Figure 5:**
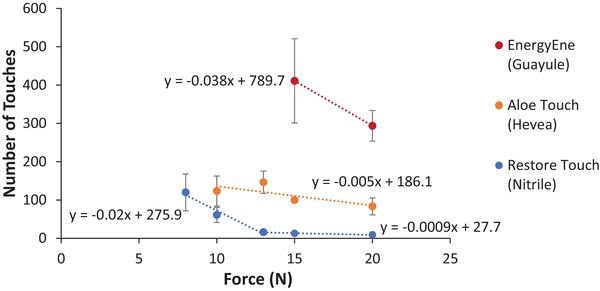
Number of touches to glove failure versus applied force ± standard error for Hevea latex, synthetic nitrile latex with colloidal oatmeal, and guayule latex. Each value is the mean of 3 gloves + standard error. Note: some error bars are smaller than the symbol height.

One of the adjustable settings on the N‐GAD is the force the sandpaper roller applies to the prosthetic hand.^[^ ^11^ ^]^ Altering this setting dramatically changes the number of touches to glove failure. However, durable gloves may take too long to test using the force required for assessing flimsy gloves (**Figure** **5**). Identifying trends in break times versus applied force provides a point of reference for testing gloves on the N‐GAD.

It should be noted that not all glove types were tested at all forces, as the number of touches to failure increased to rather extreme time periods as the force decreased (consecutive touches are ≈6 s apart) (Figure 5). Guayule latex gloves were not tested at forces lower than 15 N due to extremely long times until failure (over an hour per glove at a minimum). Hevea latex gloves were also not tested below 10 N for the same reason, with break times reaching over 26 min per glove.

The separation of relative durability of the different glove materials was maintained at the different forces (Figure 5) and the natural latex gloves exhibited a negative linear relationship of force and number of touches to break. Restore Touch nitrile gloves had two distinct trendlines: the one with a slope of −0.2 is relevant to glove testing. The second plottable trend line with a slope of −0.0009 indicates that these gloves basically could not withstand touches with any force over 13 N. Plotting such trends will aid with future N‐GAD studies in which the force must be adjusted from its default setting, and provides context for the data collected in this study at the default force of 15 N.

The unit for applied force used in this study is Newtons rather than Pascals because Newtons are a measure of force that is independent of thickness. A Pascal is derived by dividing the force in Newtons by the area of the test sample, usually in square meters. Since glove thicknesses vary among materials and brands (Figure 3), Newtons were the more appropriate unit of force for the N‐GAD. Although some ASTM glove standards include Newtons, most use Mega‐Pascals, and reported forces are highly affected by film thickness.

### 3.5 Material Properties

**Figure 6:**
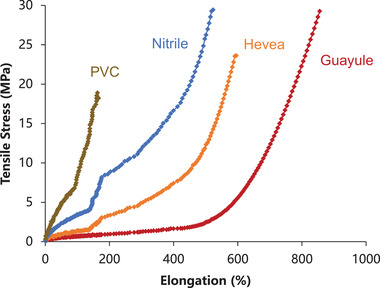
Tensile stress versus percent elongation for PVC, nitrile, Hevea, and guayule gloves.

Natural latex gloves are much more comfortable for the wearer due to their superior mechanical properties. As shown by the stress–strain curve (**Figure** **6**), the natural latex gloves, Hevea and guayule, are more elastic than synthetic gloves (PVC and a high‐quality purple nitrile glove), with guayule being the most stretchable. The point at which the plots end is where the testing dumbbell broke in two. The nitrile and guayule glove films were of similar strength, and this is generally the case when high quality nitrile gloves are tested. The modulus (effectively, softness) difference is apparent in the vertical plain of these curves: the lower the number, the greater the softness.

**Figure 7:**
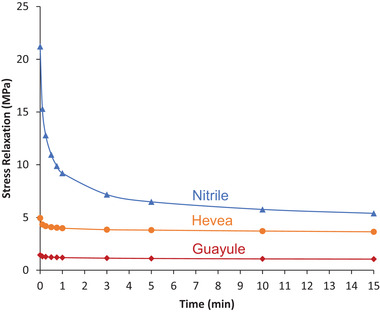
Stress relaxation over a time of 15 min for nitrile, Hevea, and guayule latex gloves.

The stress relaxation force reflects the resistance of the glove to deformation (**Figure** **7**). Thus, the nitrile glove, when first donned, is very stiff and requires substantial hand energy for several minutes as the glove warms and softens. It remains more resistant to hand movement than the two natural latex gloves. Neither of these changes much over time. The energy required to stretch guayule latex films is very low compared to other materials (Figure 7). This makes for easier donning and more comfortable wear, and it fits like a “second skin”.

**Figure 8:**
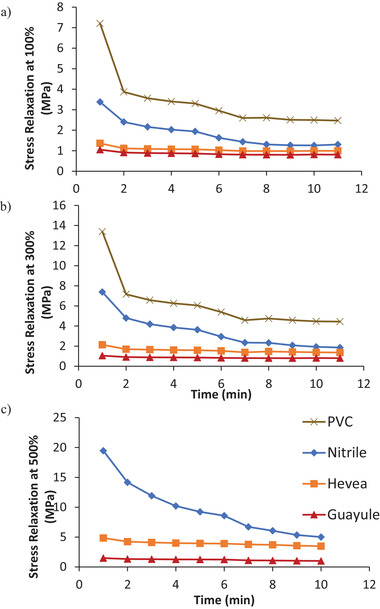
Stress relaxation for PVC, nitrile, Hevea, and guayule latex gloves at a) 100% elongation, b) 300% elongation, and c) 500% elongation. PVC is not included in the 500% elongation graph because it was unable to elongate to 500% without breaking.

When stress relaxation was determined at three elongations to compare PVC, nitrile, Hevea, and guayule, the synthetic gloves were much stiffer than the natural gloves during the first few minutes of testing (**Figure** **8**). Although PVC and nitrile substantially softened during repeated manipulation, PVC remained the stiffest glove throughout the trial, followed by nitrile, Hevea, then guayule. PVC was able to reach 300% elongation during the stress relaxation tests but reached less than 200% elongation in the tensile stress test. This reflects the presence of manufacturing inconsistencies among the PVC glove samples (Figure 6).

**Figure 9:**
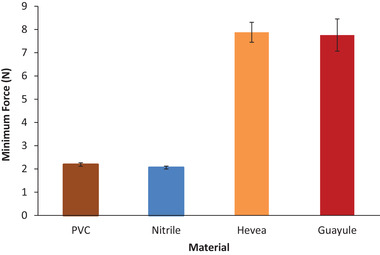
Average minimum force require to initiate a tear, in Newtons ± standard error for PVC, nitrile, Hevea and guayule examination gloves.

Natural latex gloves were more tear resistant than nitrile gloves and other synthetics (**Figure** **9**). This is because natural rubber forms crystallites when the material is stretched, and the polymers align. This phenomenon, stress–strain crystallization, is a main cause of the better performance of natural rubber than synthetic elastomers in many applications.^[^ ^12^, ^13^, ^14^, ^15^ ^]^ When material failure begins, cracks do not propagate beyond the first crystallite they encounter. Thus, crystallites block crack propagation and inhibit tearing.^[^ ^16^ ^]^

### 3.6 ASTM Standards

ASTM provides standard specifications for surgical gloves as well as examination gloves.^[^ ^3^, ^4^ ^]^ However, depending on the specific material, these minimum physical requirements may be much lower than the general standards for surgical and examination gloves. For instance, synthetic gloves such as PVC, polychloroprene, and nitrile that are typically used as examination gloves all have minimum tensile strength and ultimate elongation requirements less than that of the standard for general Type II unaged synthetic examination gloves (**Table** **5**).^[^ ^17^, ^18^, ^19^ ^]^ Polychloroprene and nitrile gloves are also held to a lower standard in terms of thickness, with their minimum requirement of 0.05 mm being less than the general exam glove minimum requirement of 0.08 mm (Table 5).

**Table 5:** ASTM physical requirements for examination and surgical gloves versus specific glove materials. Type I gloves are composed of natural rubber latex, and Type II gloves are composed of rubber cement or synthetic rubber latex

Standard Specification Title	Designation	Type	Thickness [mm]	Before Aging		After Accelerated Aging	
				Tensile Strength [MPa minimum]	Ultimate Elongation [% minumum]	Tensile Strength [MPa minimum]	Ultimate Elongation [% minimum]
Rubber Examination Gloves	D3578‐19	I	0.08	18	650	14	500
		II	0.08	14	650	14	500
Rubber Surgical Gloves	D3577‐19	I	0.10	24	750	18	560
		II	0.10	17	650	12	490
Poly(vinyl chloride) Gloves for Medical Application	D5250‐19	II	0.08	11	300	–	–
Nitrile Examination Gloves for Medical Application	D6319‐19	II	0.05	14	500	14	400
Polychloroprene Examination Gloves for Medical Application	D6977‐19	II	0.05	14	500	14	400

## 4 Discussion

Our data prove that, in general, natural latex gloves are more durable than synthetic ones and so provide a more effective barrier against pathogens and toxins. Their mechanical properties also make them a more comfortable and less tiring glove to wear.^[^ ^20^ ^]^ For synthetic PVC and nitrile gloves, the more movements the wearer makes, the more resistance the glove poses to such movements, unlike guayule that poses little resistance (Figure 6).

Among the surgical gloves, natural latices tended to outperform synthetics. The poorer durability of the natural latex Triumph Micro surgical glove compared to the Triumph Green one (Figure 2) was not due to a thickness difference (Figure 3), so probably reflects differences in the manufacturing protocols used to make them. This raises a serious concern about the quality control of their manufacturing processes because every user expects reproducible performance from different examples of a specific glove and brand.

The large disparity in the average number of touches to failure for the three nitrile glove brands (6.8 for Vglove, 11.2 for Restore Touch, and 88.8 for U.S. Medical Glove) indicates that some imported gloves are seriously substandard. These gloves have similar thickness (Figure 3), so Vglove and Restore Touch either have serious manufacturing issues, they are not made of 100% nitrile, or have a high loading of diluent filler(s). In addition, the average cuff thicknesses of these two glove types fell below the minimum ASTM required thickness of 0.05 mm for unaged nitrile gloves (Table 5).^[^ ^19^ ^]^ Similarly, the Safeko brand PVC gloves, which are also synthetic, failed to meet the ASTM minimum ultimate elongation of 300% for unaged PVC gloves (Table 5), breaking below 200% (Figure 6). This raises serious concerns over the safety of these medical gloves, which claim to meet ASTM standards. When healthcare workers use gloves, they rely on them to act as physical barriers against disease transmission. Cheap synthetic gloves are cheap for a reason, and it is clearly important to avoid purchasing them even when they are dumped on the U.S. market. Saving a few dollars by acquiring unsafe gloves can cost people their health and even their lives. The authors encourage all glove manufacturers to create high‐quality products that will protect the public from exposure to harmful pathogens.

From the thickness tests, it may initially appear that the solution to the issue of glove durability is to create thicker gloves. However, thicker gloves are also stiffer and reduce the tactile sensitivity of the wearer's hands. Both qualities are undesirable for medical gloves because dexterity is essential for delicate surgeries and other medical procedures, and thicker gloves may hinder the performance of the healthcare provider and lead to undesirable patient outcomes.

The lack of control U.S. users have over glove quality, and the disruption of supply chains experienced during the corona virus 2 (SARS‐CoV‐2) (COVID‐19) pandemic, have led to some onshoring of nitrile glove manufacturing, and increasing emphasis on raw material manufacture, including production of natural rubber and natural rubber latex within our borders.^[^ ^21^ ^]^

It is worth noting that properly leached (washed), polymer coated, unpowdered natural latex gloves made from latex tapped from tropical rubber trees can be used safely by people who do not already have Type I latex allergy.^[^ ^22^ ^]^ Since most natural latex gloves imported into the U.S. market (at least before the COVID‐19 pandemic), are properly leached and contain very little soluble protein (< 50 µg g^−1^, see ASTM D6699) it seems more likely that a user would contract a disease through synthetic glove breakage than have a dangerous anaphylactic reaction to residual proteins in a natural latex glove.^[^ ^23^ ^]^ However, although data is amassing proving that COVID‐19 has impelled the manufacture and import of cheap, poor quality synthetic gloves there is, as yet, no information on whether any natural latex glove manufacturers have chosen to lower their costs by taking their leaching step out again. If any take this irresponsible approach, a new wave of Type I latex allergy sensitization would occur. One dental dam manufacturer does not leach their natural latex dams, so this concern is not without precedence.^[^ ^24^ ^]^ Normal detergents in hot or cold water do not effectively extract entrained proteins remaining in unleached, fully cured natural latex films, but human fluids can do this during medical or dental procedures. Leaching is only effective during manufacture when applied to partially cured products. Because the findings of our glove study are imperative to the health and safety of healthcare works, a table summarizing the authors’ recommendations has been included (**Table** **6**).

**Table 6:** Author recommendations for healthcare workers. The quality of gloves was ranked using the following scale: Excellent, Good, Fair, Poor, Very Poor. Please note that examination and surgical gloves are *not* directly comparable in terms of quality rating

Brand	Material	Intended Use	Quality Rating
Vglove	Nitrile	Examination	Very Poor
Restore Touch	Nitrile	Examination	Poor
US Medical Glove	Nitrile	Examination	Excellent
Safeko	PVC	Examination	Very Poor
Aloe Touch	Hevea Latex	Examination	Poor
Triumph Green	Hevea Latex	Surgical	Good
Triumph Micro	Hevea Latex	Surgical	Fair
Neolon	Polychloroprene	Surgical	Fair
Sensicare Micro	Polyisoprene	Surgical	Good
EnergyEne	Guayule Latex	Surgical	Excellent

## 5 Conclusions

Examination and surgeon's gloves are extremely variable in durability, when tested dry and in air, even within a specific elastomeric material, indicating fundamental flaws in manufacturing protocols, quality control, and inspection rates. Unexpectedly flimsy gloves pose a threat to the health and wellbeing of all wearers, their patients, and their colleagues. Also, medical gloves are not solely used in dry environments and additional research will include testing durability under wet conditions, such as in water, ethanol, and phosphate buffered saline to simulate more realistic user environments.^[^ ^6^ ^]^ Durability under these conditions may be poorer than in air. It will be important to determine any correlation between wet and dry glove durability before an ASTM durability standard can be proposed. Overall, gloves made from guayule latex, an allergy‐safe domestic source of alternative natural rubber latex were more durable than the other gloves tested, including those made from Hevea latex.^[^ ^22^ ^]^ Guayule gloves (and condoms) have previously been demonstrated to be effective barriers against viruses, including the φX174 virus, which, with a diameter of 27 nm, is smaller than the smallest known human pathogenic virus.^[^ ^25^ ^]^ This indicates that guayule latex gloves are also effective barriers against larger pathogenic viruses.

Guayule latex is as strong as nitrile and as tear resistant as Hevea latex while also having a more comfortable, lightweight feel that allows the user to almost forget they are wearing gloves. Compared to other products such as the FlexiPalm, guayule latex gloves have similar features such as an inconspicuous and comfortable design, but guayule gloves have the added benefit of providing a barrier for the entire hand, rather than just the palm.^[^ ^26^ ^]^ Although the palm may be the main transmission point for pathogens, a hand that is fully covered is fully protected from transmitting or being contaminated by harmful bacteria and viruses. Guayule latex gloves are currently not produced on a commercial scale. However, examination, surgical, and radiation attenuation guayule latex gloves have been prototyped by multiple companies, tested by consumer groups, and consistently judged best in class.

Guayule latex can address the need for domestic production of gloves to resolve supply chain and quality issues. A shift back from synthetic latices, which are made from petroleum, to natural latices made from plants, would greatly reduce the carbon footprint of the medical glove industry as a whole and biologically and geographically diversify the natural rubber supply.

## 6 Experimental Section

### Glove Durability Tests

A variety of gloves were tested in random order on the N‐GAD and the C‐GAD, and the results were compared. Both surgical and examination gloves were tested, and the glove materials included natural latex, nitrile, polychloroprene, and polyisoprene. To ensure that the results from each glove tester were comparable, both the number and size of each glove type were kept as consistent as possible between the C‐GAD and the N‐GAD. It should be noted, however, that glove sizes 6 and 6.5 were too small for the prosthetic hand of the current N‐GAD, so size 7 was used instead. In addition, while the N‐GAD was capable of testing both left and right‐handed gloves due to the lack of a thumb on the prosthetic hand, the C‐GAD was only capable of testing right‐handed gloves. Therefore, due to limited availability of larger‐sized polychloroprene gloves, sizes 6, 6.5, and 7 were tested using the C‐GAD. For both devices, the default microcontroller settings were used to maintain consistency, including an applied force of 15 N and a motor speed of 3.5 mm s^−1^ that supports a touching interval of approximately one touch every 6 s. The data collected included the time and number of touches until the glove was punctured.

A larger array of gloves was then tested using the N‐GAD in order to compare glove materials and brands. The tested gloves included three types of nitrile gloves (Vglove, Restore Touch, and U.S. Medical Glove; all examination gloves), three types of Hevea latex gloves (Aloe Touch; examination, Triumph Green; surgical, and Triumph Micro; surgical), one type of polyisoprene glove (Sensicare Micro; surgical), one type of polychloroprene glove (Neolon; surgical), one type of PVC glove (Safeko; examination), and one type of guayule latex glove (EnergyEne; surgical). Examination gloves were sizes medium and large, and surgical gloves were sizes 7 and 8. Depending on availability, either three or five replicate gloves were tested from each type. The time and number of touches until the glove was punctured were recorded, as well as the force used.

### Thickness Measurements

Four thickness measurements were collected for three gloves of each type, using an electron caliper: three around the cuff and one at the fingertip. The third (ring) finger was selected for fingertip thickness sampling because it is one of the fingers that meets the rough surface of the drum on the N‐GAD. Fingertip thickness measurements were performed after durability testing because these measurements required the removal of the glove finger, effectively destroying the glove.

### Force Tests

Three samples of nitrile, Hevea latex, and guayule latex examination gloves were tested at forces of 8, 10, 13, 15, and 20 N to determine the correlation between the force applied by the N‐GAD and the number of touches until the glove failed and validate the use of higher forces for thicker gloves. The most durable gloves were not tested at the lowest force levels, however, due to extremely long wait times until glove breakage.

### Guayule Gloves

Guayule gloves were made one at a time, as described for guayule radiation attenuation gloves except that no Bi_2_O_3_ filler was added.^[^ ^27^ ^]^ The same xanthate‐based curing package was used that prevents the contact reactions and Type IV skin allergies often caused by the traditional carbamate, thiuram and thiazole chemical cross‐linking accelerators.^[^ ^28^ ^]^ Thus, guayule latex gloves are circumallergenic because they avoid Type I systemic reactions and Type IV and contact allergic reactions.

### Mechanical Tests

Tensile measurements according to ASTM D412 were used to compare the performance of guayule gloves and gloves made from tropical natural latex, nitrile, and polyvinyl chloride.^[^ ^29^ ^]^ Five dumbbells were cut using Die D from glove films (CCSI, Akron, OH, USA). Sample tensile properties were determined using a tensiometer (model 3366, Instron, Norwood, MA, USA) with a 50 N static load cell (model 2530–50N, Instron), equipped with a contact extensometer (model 3800, Epsilon Tech. Corp., Jackson, WY, USA). Tensile strength (stress), elongation to break, and modulus at 500% strain) were derived using Bluehill v. 2.26 software (Instron, Norwood, MA, USA). Stress relaxation was measured by elongating a sample to the desired strain (100%, 300%, or 500%), and recording the decrease in stress over a 15 min period. Tear strength was determined according to ASTM D624 using notched dumbbells and the tensiometer described above.^[^ ^30^ ^]^ Tear strength was measured by pulling the notched test sample apart and measuring the minimum amount of force required to initiate the tear.

### Statistical Analysis

One‐ and two‐way analyses of variance (α = 0.05) were performed using the statistical software JMP 14. Except for the direct comparison of the C‐GAD and N‐GAD, extreme outliers were removed from the data to ensure that the mean value accurately represented the data set. A student's *t*‐test was also performed on individual data gathered using the N‐GAD so that specific glove‐to‐glove variability could be identified.

## Conflict of Interest

K.C. is the CEO of EnergyEne, the company that lent lenthe N‐GAD for use in this study and provided the prototype guayule latex gloves. The other author declares no conflict of interest.

## Data Availability

The data that support the findings of this study are available from the corresponding author upon reasonable request.

## References

[1] L. A. Greenawald , K. C. Hofacre , E. M. Fisher , J. Occup. Environ. Hyg. 2020, 17, 398. 32658631 10.1080/15459624.2020.1784426 PMC10015298

[2] M. S. Albin , L. Bunegin , E. S. Duke , R. R. Ritter , C. P. Page , Crit. Care Med. 1992, 20, 170. 1737454

[3] ASTM D3577‐19 Standard Specification for Rubber Surgical Gloves. *ASTM International*, 2019.

[4] ASTM D3578‐19 Standard Specification for Rubber Examination Gloves, *ASTM International*, 2019.

[5] ASTM D5151‐19 Standard Test Method for Detection of Holes in Medical Gloves. *ASTM International*, 2019.

[6] R. Michel , K. Cornish , presented at the International Latex Conference , Fairlawn, OH, United States 2015.

[7] S. Thomas , E. Aldlyami , S. Gupta , M. R. Reed , S. D. Muller , P. F. Partington , Arch. Orthop. Trauma Surg. 2010, 131, 455. 20607254 10.1007/s00402-010-1146-8

[8] M. H. Bardorf , B. Jäger , E. Boeckmans , A. Kramer , O. Assadian , Am. J. Infect. Control 2016, 44, 1645. 27388267 10.1016/j.ajic.2016.03.070

[9] A. Rego , L. Roley , Am. J. Infect. Control 1999, 27, 405. 10511487 10.1016/s0196-6553(99)70006-4

[10] Global Disposable Medical Gloves Market Report https://www.grandviewresearch.com/industry‐analysis/disposable‐medical‐gloves‐market, accessed: February, 2023.

[11] A. Venturini , M. Pancake , W. VanCleave , Y. Wan , K. Cornish , Inventions 2022, 7, 62.

[12] P. Junkong , Y. Matsushima , T. Phakkeeree , K. Cornish , Y. Ikeda , Rubber and Chemistry Technology 2019, 92, 388.

[13] P. Junkong , K. Cornish , Y. Ikeda , RSC Adv. 2017, 7, 50739.

[14] P. Junkong , T. Ohashi , T. Phakkeeree , Y. Sakaki , A. Tohsan , K. Cornish , I. Ikeda , Kautschuk Gummi Kunststoffe 2017, 8, 38.

[15] Y. Ikeda , J. Preeyanuch , T. Ohashi , T. Phakkeeree , Y. Sakaki , A. Tohsan , S. Kohjiya , K. Cornish , RSC Adv. 2016, 6, 95601.

[16] H. P. Zhang , J. Niemczura , G. Dennis , K. Ravi‐Chandar , M. Marder , Phys. Rev. Lett. 2009, 102, 4. 10.1103/PhysRevLett.102.245503 19659026

[17] ASTM D5250‐19 Standard Specification for Poly(vinyl chloride) Gloves for Medical Application, *ASTM International* 2019.

[18] ASTM D6977‐19 Standard Specification for Polychloroprene Examination Gloves for Medical Application, *ASTM International* 2019.

[19] ASTM D6319‐19 Standard Specification for Nitrile Examination Gloves for Medical Application, *ASTM International* 2019.

[20] P. Mylon , R. Lewis , M. J. Carré , N. Martin , S. Brown , Am. J. Infect. Control 2014, 42, 48. 24268835 10.1016/j.ajic.2013.07.009

[21] C. P. Bown , Asian Economic Policy Review 2022, 17, 114.

[22] K. Cornish , Rubber Science 2012, 25, 139.

[23] ASTM D6699‐16 Standard Practice for Sampling Liquids Using Bailers, *ASTM International* 2016.

[24] K. Cornish , G. M. Bates , J. L. Slutzky , A. Meleshchuk , W. Xie , K. Sellers , R. Mathias , M. Boyd , R. Casteñeda , M. Wright , L. Borel , Biology and Medicine 2019, 11, 1000456.

[25] K. Cornish , C. D. Lytle , J. Biomed. Mater. Res. 1999, 47, 434. 10487897 10.1002/(sici)1097-4636(19991205)47:3<434::aid-jbm20>3.0.co;2-n

[26] J. K. S. Tan , S. W. Song , J. Zeng , C. H. Lo , Bioeng. Transl. Med. 2022, 10411. 10.1002/btm2.10411 PMC9538315 36248233

[27] D. A. Ramirez Cadavid , R. R. Layman , T. Nishino , J. L. Slutzky , Z. Li , K. Cornish , Materials 2022, 15, 1184. 35161128 10.3390/ma15031184 PMC8839583

[28] R. Virdi , B. Grover , K. Ghuman , Rubber Chem. Technol. 2019, 92, 90.

[29] ASTM D412‐16 Standard Test Methods for Vulcanized Rubber and Thermoplastic Elastomers‐ Tension, *ASTM International*, 2021.

[30] ASTM D624‐00 Standard Test Method for Tear Strength of Conventional Vulcanized Rubber and Thermoplastic Elastomers, *ASTM International*, 2020.

